# Supplementary material for: Clinical Predictors of Cognitive Impairment in a Cohort of Patients with Older Age Bipolar Disorder
Source: Brain Sci. 2025 Mar 27;15(4):349. doi: 10.3390/brainsci15040349 (PMC12026280; doi:10.3390/brainsci15040349)
Supplement: Supplementary file 1 [file brainsci-15-00349-s001.zip › brainsci-3492292-supplementary.pdf]

brain sciences

Comparisons among patients without cognitive impairment, with MCI and with dementia

The subgroups of cognitively unimpaired patients, patients with MCI, and patients diagnosed with dementia did not exhibit significant differences in age, gender, BMI, or educational levels (see Table S1). Marital status showed some significant differences. Post-hoc tests revealed that, among patients without cognitive impairment, unmarried individuals were more common than divorced or widowed individuals compared to those with MCI. Similarly, married patients were more common than divorced patients among those without cognitive impairment compared to those with MCI (p_{fdr}<0.05).

Mood disorders revealed a trend toward more OS-BD/CD in MCI and dementia groups compared to cognitively unimpaired group. The prevalence of BD type 1 was similar in the cognitively unimpaired and MCI groups but lower in the dementia group. BD type 2 was most prevalent in the cognitively unimpaired group and decreased in MCI and dementia groups.

No statistically significant differences were observed in lifetime anxiety disorders (with the exception of agoraphobia, which, however, did not show significant differences at post-hoc tests) or substance use disorder.

Additionally, somatic comorbidities such as thyroid dysfunction, hypertension, type 2 diabetes mellitus, or obesity showed comparable prevalence across groups without significant differences. Dyslipidemia and vascular leukoencephalopathy were most prevalent in the MCI group, with a lower prevalence in the cognitively unimpaired group and an intermediate prevalence in the dementia group. However, while the overall difference was significant, no significant difference emerged from post-hoc tests.

The analysis of first-degree family history revealed no significant differences in the prevalence of any psychiatric disorder across groups, as well as no significant differences for mood disorders, bipolar or related disorders, or anxiety disorders.

No significant differences were noted for family history of neurodegenerative diseases overall or Parkinson's Disease. A significant difference was observed in the prevalence of family history of dementia, which was higher in the dementia group compared to cognitively unimpaired group and MCI group. Additionally, a family history of AD was more frequent in the dementia group compared to cognitively unimpaired group and MCI group. However, while higher prevalence was observed in patients with dementia compared to the other two subgroups, no significant difference emerged from post-hoc tests.

No significant difference was found in the age at onset of psychiatric symptoms. The median age at onset of mood symptoms demonstrated a trend toward significance, being comparable between the cognitively unimpaired group and the MCI group, but occurring at a later age in the dementia group. Significant differences were observed for the age at the first major mood episode, which occurred later in the MCI group and dementia group compared to cognitively unimpaired group. Similarly, the first depressive episode occurred later in MCI and dementia groups compared to cognitively unimpaired group, though the difference was not statistically significant. The first (hypo)manic episode showed a trend toward significance, with a later onset in MCI and dementia groups compared to cognitively unimpaired group. These findings suggested a general trend of later onset of psychiatric and mood episodes in individuals with cognitive impairment.

Illness duration showed a decreasing trend from cognitively unimpaired group to MCI group and dementia group, though this difference was not statistically significant. The polarity of onset as a (hypo)manic episode was slightly more common in the MCI group and dementia group compared to cognitively unimpaired group, but did not reach statistical significance. No significant differences were found in the history of psychosis and suicidal attempts across groups.

History of hospitalization showed a non-significant trend toward significance, with no hospitalizations being more frequent in MCI and dementia groups compared to cognitively unimpaired group, while single hospitalizations were more common in cognitively unimpaired group than in MCI and dementia groups, and multiple hospitalizations were more frequent in cognitively unimpaired group compared to MCI and dementia groups. Lifetime depressive episodes were significantly fewer in dementia group compared to cognitively unimpaired group, while the MCI group showed intermediate values without significant differences from the other groups. No significant differences were observed for lifetime manic episodes or lifetime hypomanic episodes. These findings suggested a trend toward a shorter illness duration and fewer depressive episodes in individuals with cognitive impairment, while other illness characteristics remained comparable across groups.

The current state of BD at the time of the first assessment showed that cognitively unimpaired patients where significantly more affected by depressive episodes than euthymia compared to subjects suffering from dementia.

The psychometric assessment highlighted significant differences across groups. CGI-S scores were comparable across all groups. However, significant differences were observed in the GAF scores, with demented patients showing the lowest score. Similarly, in the BPRS total score: statistically significant differences emerged showing an increasing trend with the lowest scores in cognitively unimpaired patients followed by patients with MCI and dementia

While no significant differences were observed in the BPRS Depression/Anxiety subscale, significant differences emerged in the BPRS Activation subscale, with scores increasing from cognitively unimpaired group to MCI and dementia groups. The BPRS Negative symptoms/Retardation subscale also showed a significant increase from cognitively unimpaired patients to those with MCI and dementia. BPRS Psychosis scores were significantly higher in the dementia group compared to the cognitively unimpaired group and the MCI group (p_{fdr}<0.05).

Table S1. Differences between patients without cognitive impairment, with MCI and with dementia. Patients were categorized into three subgroups based on the presence of a cognitively unimpaired condition, of MCI or of dementia of any type. Kruskal-Wallis test was used for comparisons of continuous variables after excluding normality using Shapiro-Wilk test. Pearson's chi-squared tests or, when appropriate, Fisher's exact test were used for comparisons of categorical variables. Differences are referred to as statistically significant as for $p < 0.05$ (shown in bold).

	**Cognitively Unimpaired (n=82)**	**MCI (n=41)**	**Dementia (n=29)**		
	**Median [IQR]**	**Median [IQR]**	**Median [IQR]**	**SMD**	***p***
**Demographic variables**					
**N**	82	41	29		
**Age**	75.00 [70.25, 79.00]	76.00 [69.00, 81.00]	78.00 [72.00, 82.00]	0.072	0.606
**Sex = M**	34 (41.5%)	11 (26.8%)	11 (37.9%)	0.208	0.282
**Schooling years (n=149)**	8.00 [5.00, 13.00]	8.00 [5.00, 13.00]	9.00 [5.00, 13.00]	0.082	0.717
**Marital status***				0.601	**0.007**
**Unmarried**	9 (11.0%)	0 (0.0%)	0 (0.0%)		
**Married**	49 (59.8%)	17 (41.5%)	18 (62.1%)		
**Divorced**	5 (6.1%)	9 (22.0%)	2 (6.9%)		
**Widowed**	19 (23.2%)	15 (36.6%)	9 (31.0%)		
**Mood Disorders**				0.450	0.069
**BD type 1**	18 (22.0%)	10 (24.4%)	3 (10.3%)		
**BD type 2**	37 (45.1%)	10 (24.4%)	10 (34.5%)		
**OS-BD/CD**	27 (32.9%)	21 (51.2%)	16 (55.2%)		
**Psychiatric comorbidities**					
**Anxiety disorder**	56 (68.3%)	29 (70.7%)	17 (58.6%)	0.170	0.537
**Panic disorder**	39 (47.6%)	18 (43.9%)	13 (44.8%)	0.049	0.919
**Generalized anxiety disorder**	26 (31.7%)	15 (36.6%)	8 (27.6%)	0.129	0.722
**Separation anxiety disorder***	15 (18.3%)	3 (7.3%)	2 (6.9%)	0.233	0.177
**Agoraphobia***	13 (15.9%)	3 (7.3%)	0 (0.0%)	0.427	0.038

**Substance use disorder***	9 (11.0%)	7 (17.1%)	7 (24.1%)	0.234	0.189
**Somatic comorbidities/features**					
**Vascular leukoencephalopathy**	25 (30.5%)	22 (53.7%)	13 (44.8%)	0.320	**0.037**
**Thyroid disease**	22 (26.8%)	11 (26.8%)	7 (24.1%)	0.041	0.957
**Hypertension**	43 (52.4%)	24 (58.5%)	11 (37.9%)	0.280	0.226
**Type 2 diabetes mellitus ***	12 (14.6%)	9 (22.0%)	4 (13.8%)	0.143	0.558
**Dyslipidemia**	30 (36.6%)	25 (61.0%)	13 (44.8%)	0.333	**0.037**
**Obesity***	9 (11.0%)	5 (12.2%)	3 (10.3%)	0.039	1.000
**BMI (n=114)**	24.77 [22.23, 27.24]	24.69 [21.76, 27.13]	22.89 [21.14, 27.68]	0.071	0.728
**First-degree family history**					
**Any psychiatric disorder**	58 (70.7%)	29 (70.7%)	20 (69.0%)	0.026	0.983
**Any mood disorders**	46 (56.1%)	24 (58.5%)	12 (41.4%)	0.232	0.310
**Any bipolar or related disorder**	13 (15.9%)	9 (22.0%)	6 (20.7%)	0.104	0.671
**Any anxiety disorder**	19 (23.2%)	9 (22.0%)	9 (31.0%)	0.138	0.640
**Neurodegenerative disease**	29 (35.4%)	15 (36.6%)	14 (48.3%)	0.176	0.456
**Any dementia**	16 (19.5%)	5 (12.2%)	11 (37.9%)	0.413	**0.030**
**Alzheimer's Disease***	7 (8.5%)	1 (2.4%)	6 (20.7%)	0.405	0.038
**Parkinson's Disease***	7 (8.5%)	4 (9.8%)	1 (3.4%)	0.171	0.667
**Age at onset**					
**Psychiatrics Symptoms**	23.00 [18.00, 46.00]	30.00 [20.00, 55.00]	35.00 [20.00, 58.00]	0.237	0.134
**Mood symptoms**	30.00 [20.00, 54.25]	30.00 [20.00, 55.00]	51.00 [30.00, 60.00]	0.316	0.071
**First major mood episode (n = 144)**	35.00 [23.00, 55.00]	51.00 [30.00, 58.50]	53.50 [36.25, 60.75]	0.401	**0.008**
**First depressive episode (n=128)**	35.00 [23.00, 52.00]	46.00 [30.00, 57.00]	51.00 [31.00, 59.00]	0.301	0.057
**First (hypo)manic episode (n=106)**	53.00 [30.50, 63.75]	59.00 [44.50, 68.00]	58.00 [52.00, 72.00]	0.370	0.076
**Illness features**					
**Illness duration**	38.50 [18.25, 53.00]	36.00 [17.00, 52.00]	25.00 [7.00, 47.00]	0.269	0.180
**Polarity onset (hypo)manic episode (n=144)**	17 (21.5%)	11 (28.2%)	7 (26.9%)	0.103	0.686
**History of psychosis (n=142)**	18 (24.0%)	11 (27.5%)	9 (33.3%)	0.138	0.638
**History of suicidal attempts (n=132)***	12 (16.4%)	4 (11.1%)	3 (13.0%)	0.103	0.840
**History of hospitalization (n=128)***				0.408	0.093

**Single**	18 (25.0%)	4 (12.5%)	2 (8.3%)		
**Multiple**	24 (33.3%)	7 (21.9%)	6 (25.0%)		
**None**	30 (41.7%)	21 (65.6%)	16 (66.7%)		
**Lifetime depressive episodes (n=146)**	3.00 [2.00, 5.00]	2.00 [1.00, 4.00]	1.00 [1.00, 3.75]	0.226	**0.015**
**Lifetime manic episodes (n=147)**	0.00 [0.00, 0.00]	0.00 [0.00, 1.00]	0.00 [0.00, 0.00]	0.226	0.633
**Lifetime hypomanic episodes (n=147)**	1.00 [0.00, 3.00]	1.00 [0.00, 1.00]	1.00 [0.00, 1.00]	0.224	0.413
**Current state***				0.553	**0.018**
**Depressive episode**	37 (45.1%)	12 (29.3%)	4 (13.8%)		
**Hypomanic episode**	10 (12.2%)	6 (14.6%)	5 (17.2%)		
**Manic episode**	1 (1.2%)	4 (9.8%)	2 (6.9%)		
**Euthymia**	34 (41.5%)	19 (46.3%)	18 (62.1%)		
**Psychometric assessment**					
**CGI**	3.00 [2.00, 4.00]	4.00 [3.00, 4.00]	4.00 [3.00, 4.00]	0.334	0.135
**GAF**	65.00 [50.00, 80.00]	60.00 [50.00, 70.00]	35.00 [25.00, 40.00]	1.528	**<0.001**
**BPRS total score (n=150)**	37.00 [31.00, 43.75]	43.00 [36.00, 48.00]	49.00 [44.00, 56.50]	0.823	**<0.001**
**BPRS Depression/Anxiety**	13.00 [9.25, 16.00]	14.00 [11.00, 16.00]	13.00 [9.00, 15.00]	0.066	0.909
**BPRS Activation**	12.00 [9.25, 16.00]	14.00 [11.00, 20.00]	16.00 [12.00, 22.00]	0.425	0.005
**BPRS Negative Symptoms/Retardation (n=151)**	6.00 [5.00, 7.00]	8.00 [6.00, 9.00]	11.50 [9.00, 14.25]	1.110	**<0.001**
**BPRS Psychosis**	6.00 [6.00, 7.75]	7.00 [6.00, 11.00]	10.00 [8.00, 14.00]	0.667	**<0.001**

BD: Bipolar Disorder; BMI: Body Mass Index; BPRS: Brief Psychiatric Rating Scale; CGI: Clinical Global Impression; GAF: Global Assessment of Functioning; IQR: Interquartile Range; M: median value; MCI: Mild Cognitive Impairment; N: frequency; OS-BD/CD: otherwise specified bipolar disorder/cyclothymic disorder; SMD: Standardized Mean Difference.
* = variables for which Fisher test was used instead of Chi-squared test
